# A stature-specific concept for uncemented, primary total hip arthroplasty: 10-year results in 155 patients using two stem shapes and modular necks

**DOI:** 10.3109/17453671003587077

**Published:** 2010-03-31

**Authors:** Georg W Omlor, Hannah Ullrich, Knut Krahmer, Alexander Jung, Günther Aldinger, Peter Aldinger

**Affiliations:** ^1^Department of Orthopaedic Surgery, University of Heidelberg; ^2^Department of Orthopaedic Surgery Paulinenhilfe, Diakonie-Klinikum, StuttgartGermany

## Abstract

**Background and purpose:**

Variations in hip anatomy limit the femoral canal fit of standard uncemented hip stems. In addition, there are still issues with leg length discrepancy and offset reconstruction, potentially resulting in impingement, dislocation, and wear. Modular stems with different shapes for femoral canal fit and multiple neck options may improve the outcome and reduce complications.

**Patients and methods:**

173 patients (190 hips) received an uncemented THA with 1 of 2 different stem shapes for canal fit and a modular neck for stature-specific hip reconstruction. Median follow-up time was 9 (7–13) years. During the follow-up period, 20 patients died (22 hips) and 12 patients (13 hips) were lost to follow-up. 155 hips were available for evaluation, including clinical and radiological outcome.

**Results:**

1 stem was revised for a periprosthetic fracture following trauma; 10 cups and 2 modular necks were revised (1 for breakage and 1 during cup revision). At 10 years, stem survival was 100%, modular neck survival was 99% (CI: 95–100), and cup survival was 94% (CI: 87–97). No leg length discrepancies were measured in 96% of cases. Offset with anatomic lateralization was achieved in 98%. Median Harris hip score was 94 (47–100) and median Merle d'Aubigné score was 16 (10–18). Relevant radiolucent lines and osteolysis were not found.

**Interpretation:**

The uncemented modular neck, dual-stem system used in this series allows accurate reconstruction of the joint by adapting the implant to the needs of the patient. This may improve the outcome of primary THA, which is supported by the results of this medium-term follow-up evaluation.

## Introduction

Leg length discrepancy, insufficient offset reconstruction, and impingement may lead to limping, dislocation, and increased wear rates (Soong et al. 2004, Widmer and Majewski 2005, Malik et al. 2007, Patel et al. 2007). One strategy to address

these problems is to improve the mechanical reconstruction of the joint by using implant designs with multiple adjustment options, e.g. prostheses with additional modular necks together with the generally used stem, head, and cup options (Helm and Greenwald 2005). This may be especially useful in situations with acetabular dysplasia, femoral deformity, and bone loss or in posttraumatic and revision THA—but also in the standard situation.

Here we describe a series with an uncemented, straight tapered stem, which has 2 different stem shapes for improved proximal canal fit and a range of modular necks, resulting in multiple reconstruction options for varus or valgus as well as anteversion or retroversion. The theoretical advantages include better reconstruction of anatomical offset and improved range of motion, and reduced risk of dislocation (Dennis and Lynch 2005, Widmer and Majewski 2005, Garcia-Rey et al. 2008). On the other hand, modularity with several metal-to-metal connectors in the implant may result in a higher risk of mismatch, fretting, and corrosion (Collier et al. 1992, Bobyn et al. 1994, Cook et al. 1994, Urban et al. 1994) or mechanical failure (Collier et al. 1992, Barrack et al. 1993, Helm and Greenwald 2005).

The aim of our study was to determine the medium- to long-term outcome of uncemented THA with a modular dual-stem system. We evaluated the clinical and radiographic outcome of a consecutive multisurgeon series of 173 patients who received 190 hips.

## Patients and methods

### Implants

**Figure 1.**
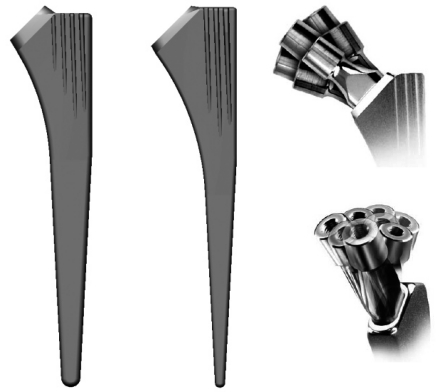
Profemur E dual stem with standard and “plus” version, and several neck options due to modular neck design.

The Profemur E/EHS implant system (European Hip System; Wright Medical Technology Inc., Arlington, TN) is an uncemented, straight modular stem made of Ti6Al4V alloy (titanium, aluminium, and vanadium) with a grit-blasted surface finish and an additional proximal hydroxyapatite coating. 10 stem sizes in a standard and “plus” version (proximally 1 cm longer) are available. The standard stem is designed for the stove-pipe femur (Dorr type B/C), whereas the plus stem is designed for the champagne-fluted type of femur (Dorr type A) (Dorr et al. 1993). 18 neck options are available from 5 different neck designs in a short and long version. The 5 neck versions include a neutral neck, an 8° angled neck for varus or valgus, an 8° angled neck for anteversion or retroversion, a 15° angled neck for anteversion or retroversion, and a neck with a combination of 4° for varus or valgus and 6° for anteversion or retroversion. With short, medium, and long heads, a 17.5-mm variation in leg length is possible (Figure 1).

The Harris Galante I cup (Zimmer, Warsaw, IN) and the EHS cup (Wright Medical) were used as the acetabular component. Both cups have a press-fit, hemispherical design made of titanium with mesh or grit-blasted surfaces; they were also fixed with screws in this study. For all hips, a polyethylene insert and a 28-mm Biolox ceramic head was used (Ceramtec, Plochingen, Germany).

### Operative technique and postoperative management

A transgluteal, lateral Bauer approach with the patient in the supine position was used in all cases. The acetabular bone was prepared using a hemispherical powered reamer of increasing size, and an attempt was made to preserve the subchondral bone in the acetabular roof. Cups were positioned in press-fit technique and also fixed with screws. The femoral canal was prepared using a canal finder and a pneumatic hammer with chip tooth broaches of increasing size. Implant type, size, neck, and head were selected according to preoperative planning and intraoperative stability and impingement testing.

Preoperative planning was done on a standard AP view of the hip with internal hip rotation to achieve an orthogonal view of the femoral neck, which neutralizes neck anteversion. If this was not possible due to the loss of rotation in the hip, the patient received an additional AP view in abdominal position with elevation of the contralateral hip, which allowed better internal rotation. Standard planning templates, provided by the prosthesis manufacturer, were used. Hence, templates were selected that would result in a joint reconstruction with equal leg length and offset and lateralization according to the opposite hip, with reconstruction of the Ménard Shenton line. The choice between the two stem versions (“standard” and “plus”) was made according to the best proximal canal fit with close contact with the medial cortex.

Perioperative single-dose antibiotic prophylaxis was used (2 g Cephazolin) as well as postoperative NSAID administration for the prevention of heterotopic ossification (50 mg diklofenac or 600 mg ibuprofen twice a day for 2 weeks). Postoperative full weight bearing was encouraged in all patients. Low-molecular-weight heparin was administered for 6 weeks together with compression stockings.

### Patients and assessment

**Figure 2.**
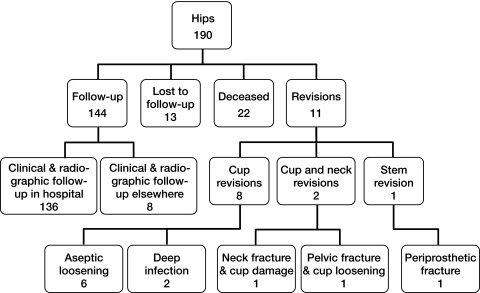
Distribution of hips at final follow-up.

This study involved a consecutive series of 173 patients with 190 primary THAs implanted between January 1993 and December 1997 (Table 1). Median follow-up was 9 (7–13) years. During the follow-up period, 20 patients died (22 hips), and 12 patients (13 hips) were lost to follow-up (Figure 2). For the 20 patients who had died, data were available to show that no hip revision had been performed. Follow-up data were obtained for 155 hips. Of those, 136 hips were clinically and radiologically assessed at our institution. 19 were examined by their local orthopedic surgeon, including standard radiographs that were sent to our institution. The clinical assessment included limp, leg length discrepancy, range of motion, and pain. In addition, a standardized questionnaire was used to assess the Harris hip score (Harris 1969), the Merle d'Aubigné score (D'Aubigne and Postel 1954), the activity score (Devane et al. 1997), and the Charnley class (Charnley 1972). Patients assessed their pain in the operated hip at the time of follow-up, on a visual analog scale (range 0–10). Radiographs were examined for radiolucent lines by 2 independent, experienced orthopedic surgeons (cup: according to DeLee and Charnley (1976); stem: according to Gruen et al. (1979)). Also, osteolysis was determined as regional, progressive bone changes on serial radiographs. Femoral cortical hypertrophy was assessed according to Gruen et al. (1979), polyethylene wear with head decentralization according to Pieringer et al. (2003), heterotopic ossifications according to Brooker et al. (1973), stem alignment according to Ebied et al. (2005), inclination angle of the cup according to Ackland et al. (1986), and offset and lateralization according to McGrory et al. (1995).

**Table 1.** Diagnoses and demographics of the 173 patients (190 hips)

Sex^**a**^	
Female	119 (63)
Male	71 (37)
Side^**a**^	
Right	106 (56)
Left	84 (44)
Diagnosis^**a**^	
Osteoarthritis	101 (53)
Developmental dysplasia of the hip	65 (34)
Avascular necrosis	11 (6)
Posttraumatic osteoarthritis	3 (2)
Others	10 (5)
Age	
Women	66 (44–83)
Men	66 (37–76)
Body mass index	
Women	27
Men	28
^**a**^Number of hips (%).	

### Statistics

Survival curves were estimated by the Kaplan-Meier method with 95% asymptotic confidence intervals (CIs). The start point of survival times was the operation date and the endpoint was the revision. Statistical analysis was performed using SAS version 9.1.3 for Windows. All results were regarded as hypothesis-generating.

## Results

### Femoral implants used for joint reconstruction

The standard version of the stem was used in 59% of the cases, and the plus version in 41%. Long necks were used in 71%, and short necks in 29%. Different neck versions were used in the following percentage: 55% necks with 8° varus/valgus; 15% neutral necks; 7% necks with 8° anteversion/retroversion; 9% necks with 15° anteversion/retroversion; and 14% necks with a combination of 4° anteversion/retroversion and 6° varus/valgus. Short heads were used in 44% of cases, medium heads in 27%, and long heads in 29%.

### Revisions

There were 11 revision surgeries for all 190 hips. In 1 hip, the stem was revised following a periprosthetic fracture with adequate trauma 12 years postoperatively. In 10 hips (5%), the cup was revised. 6 cups (3%) were revised for aseptic loosening and 2 cups were revised for deep infection without neck revision. In 2 other hips, the cup was revised together with the modular neck. Here, 1 patient sustained a fracture of the pelvic ring with cup loosening. The neck was exchanged for a different neck to improve anatomical reconstruction in the revision case. In the second patient, a stress fracture of the modular neck (short neck with 8° varus) occurred at a laser mark on the neck close to the cone. Due to additional polyethylene liner damage and damage to the fixation of the liner, both the cup and neck were revised.

### Survival estimate (Figure 3)

The endpoint “all revisions” showed a low annual failure rate and a 10-year survival rate free of any revision of 94% (95% confidence interval (CI): 89–98). This survival rate of 94% dropped to 88% (CI: 71–93) after 12.3 years because of a periprosthetic fracture of the femur. Immediately at final follow-up (12.8 years), the calculated survival rate dropped to 44% (CI: 1–86) because 1 event (cup revision) occurred at the final follow-up when only 1 other hip was still under follow-up. Thus, this survival rate at final follow-up is not reliable. Survival with the endpoint “all cup revisions” was 94% (CI: 87–97) at 10 years. Because of 1 cup revision at final-follow-up, the cup survival rate then dropped to 47% (CI: 1–88), which was not reliable (see above). Survival with “all neck revisions” as the endpoint was 99% (CI: 95–100) at 10 and 12.8 years. Survival with the endpoint “stem revision” was 100% after 10 years. At 12.3 years, there was 1 stem revision due to a periprosthetic fracture with adequate trauma. Hence, the stem survival rate dropped to 94% (CI: 65–99), which was not reliable since only 16 other hips were under follow-up.

**Figure 3.**
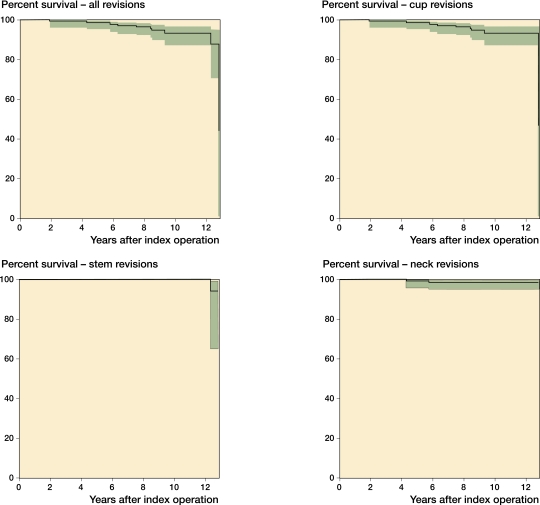
Kaplan-Meier curves with 95% confidence intervals demonstrate percent survival for different endpoints. 10-year survival with confidence intervals: all revisions, 93.5% (89–98); cup revisions, 93.5% (87–97); stem revisions, 100%; neck revisions, 98.8% (95–100).

### Clinical results (Table 2)

Complete questionnaires were obtained from 135 patients (corresponding to148 hips). 96% (CI: 91–98) of the patients were extremely satisfied or satisfied with their procedure. 79% (CI: 71–86) of the patients had a walking distance of more than 1 kilometer and 48% (CI: 39–57) had no problems in walking several kilometers. No limping was found in 82% (CI: 74–88) of the hips; obvious limping was found in 4% (CI: 2–9)—but this was caused by external factors and co-morbidities. No leg length discrepancies were measured in 96% of the patients (CI: 91–98); 2% (CI: 0–6) had a leg length difference of less than 0.5 cm and another 2% (CI: 0–6) had a leg length difference of 0.5–1.5 cm. Patients with a leg length difference of 0.5–1.5 cm had severe dysplasia and had had trochanteric or pelvic osteotomies preoperatively.

**Table 2.** Summary of clinical results

Harris hip scores, median (range)	
All hips (n = 148)	93.5 (47–100)
Harris-Galante I cup (n = 40)	93 (47–100)
EHS cup (n = 108)	94 (61–100)
Charnley A (n = 31)	94 (65–100)
Charnley B (n = 49)	95 (47–100)
Charnley C (n = 68)	88 (50–100)
Score distribution, % (n)	
Excellent (90–100)	60.1 (89)
Good (80–89)	18.3 (27)
Moderate (70–79)	14.2 (21)
Poor (< 70)	7.4 (11)
Merle d'Aubigné Scores, median (range)	
All hips	16 (10–18)
Harris-Galante I cup	16 (11–18)
EHS cup	17 (10–18)
Score distribution, % (n)	
Excellent (16–18)	52 (77)
Good (14–15)	16 (23)
Moderate (12–13)	21 (31)
Poor (< 11)	12 (17)
Pain on visual analog scale, % (n)	
No pain (0)	78 (116)
Slight pain (1–3)	12 (18)
Moderate pain (4–6)	9.4 (14)
Severe pain (7–10)	0 (0)
Activity scores, % (n)	
Grade 5 (heavy work/sports)	0 (0)
Grade 4 (moderate work/sports)	1.3 (2)
Grade 3 (easy work/sports)	61 (90)
Grade 2 (mostly sitting)	34 (50)
Grade 1 (confined to bed)	4.1 (6)

### Radiographic findings (Figure 4)

#### Stem.

Radiolucent lines were found in 4 hips (3% (CI: 1–6)) and they were less than 2 mm thick. Proximal femoral osteolysis was found in 3 hips (2% (CI 0–6)) and was less than 1 cm^2^ in size in all cases. For both criteria, no progression over time was detectable on serial radiographs. Femoral cortical hypertrophy was found around the distal stem in 30% (CI: 23–38) of cases. In 2 hips, (1% (CI: 0–5)) varus alignment of the stem was found, whereas 2 other hips (1% (CI: 0–5)) showed valgus alignment. In all cases, no migration was detected on serial radiographs. Due to medialization of the cup by a median of 5 mm, femoral offset was increased from 4.2 (2.2–6.1) cm preoperatively to 4.8 (2.5–6.9) cm postoperatively to achieve anatomic lateralization. In 98% (CI: 94–100) of the cases, correct lateralization of the hip joint was achieved.

**Figure 4.**
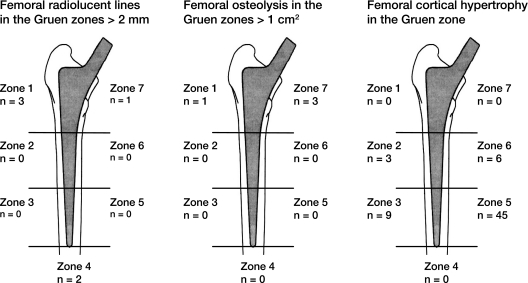
Localization and incidence of femoral radiolucent lines, femoral osteolysis, and cortical hypertrophy.

#### Acetabulum

Radiolucent lines and osteolysis around the acetabular component were found in 15% (CI: 10–22) and 3% (CI: 1–6) of all hips, respectively (Table 3). The median inclination angle of the cups was 43° (23–72) with 18% (CI: 12–25) < 40°, 62% (CI: 54–70) 40°–45°, and 20% (CI: 14–27) > 45°. Polyethylene wear with head decentralization was found in 19% (CI: 13–26) of all the acetabular components. 37% (CI: 23–53) of Harris-Galante I cups and 13% (CI: 7–20) of EHS cups showed decentralization. Heterotopic ossification was found in 30% (CI: 23–38) of all hips with 19% grade I, 7% grade II, 4% grade III, and 1% grade IV.

**Table 3.** Radiolucent lines and osteolysis of the two different acetabular components

	Harris-Galante I cup (n = 43)	EHS cup (n = 112)
Radiolucent lines		
Zone I	0	10
Zone II	0	6
Zone III	2	15
Osteolysis		
Zone I	1	3
Zone II	1	2
Zone III	1	0

#### Complications

Intraoperative complications included 1 femoral fissure and 1 trochanteric fracture, which did not require further surgical intervention. Postoperative complications were heparin-induced thrombocytopenia with pulmonary embolism (n = 1), deep vein thrombosis with pulmonary embolism (n = 2), postoperative seroma with early surgical intervention (n = 1), and abduction deficit with surgical tendon release 4 months after arthroplasty (n = 1).

No early dislocations occurred. However, 2 hips had dislocations due to cup migration with aseptic loosening and had to be revised (hip 1: Harris-Galante I cup, dislocation due to aseptic loosening after 9 years; hip 2: EHS cup, dislocation due to aseptic loosening after 6 years).

## Discussion

In the past, two main factors affecting the long-term survival of uncemented implants have been identified: (1) primary stability with stable bone-implant interlock and minimal micromotion, and (2) secondary osseointegration for long-term stability (McNally et al. 2000, D'Antonio et al. 2001, Grubl et al. 2002). Most uncemented implants available today comply with these requirements. However, problems with dislocation, osteolysis, wear, leg length discrepancy, and offset reconstruction remain challenging (Widmer and Majewski 2005, Maheshwari et al. 2007, Patel et al. 2007).

A wide range of femoral anatomies has to be taken into account in the design of femoral stems, particularly in the proximal femoral canal area (Dorr et al. 1993, Husmann et al. 1997). Femoral canal shape can vary between a champagne-fluted and a stove-pipe configuration; thus, the 2 stem versions were used to improve proximal fit (Noble et al. 1988, Dorr et al. 1993). In stove-pipe femur configurations, which are typically found in older women with osteoporotic bone, the standard version was used, whereas the “plus” version was used for the more champagne-fluted femurs most often found in younger males. Since extended contact areas in the metaphyseal zone allow better reduction of micromotions and, consequently, extended osteointegration of the implant (Sandborn et al. 1988, Galante and Jacobs 1992), this might have contributed to the absence of aseptic stem loosening in our study, as proximal canal fill with full contact to the calcar region was achieved.

The modular neck system can make femoral reconstruction easier, since femoral replacement is divided into 2 steps. In the first step, stable stem fixation can be achieved regardless of joint reconstruction parameters; in the second step, the anatomical reconstruction of the joint is done by selecting the appropriate neck. This means that multiple neck options permit correct offset reconstruction and better adjustment of retroversion/anteversion and leg length. Together with other factors, this may explain the good anatomical reconstruction in this series, with the absence of dislocations (Soong et al. 2004) and extensive osteolysis or wear even though we used conventional polyethylene, gamma-sterilized in air. Improved reconstruction of joint parameters may have contributed to the low incidence of femoral osteolysis and the good survival rates.

In the past, major concerns about modular THA have been raised, due to the risk of mismatch, fretting, and corrosion (Collier et al. 1992, Bobyn et al. 1994, Cook et al. 1994) or mechanical failure (Collier et al. 1992, Barrack et al. 1993, Helm and Greenwald 2005). In our study, 2 modular necks were revised. In one case a loose cup was revised, and for better reconstruction the neck was exchanged. In this case modularity made the revision easier, so modular necks can offer an advantage in revision surgery. The second case had a neck fracture without adequate trauma 6 years after primary THA. The fractured neck was a short one with 8° varus. Later analysis by electron microscopy revealed a fatigue fracture of the neck beneath the stem-neck connection, which was related to a laser mark on the neck. The localization of the laser mark was changed and to date no other neck fractures have occurred. Further mechanical in vitro testing was done, including other neck versions that are exposed to higher mechanical demands (i.e. medium and long varus necks and necks with additional anteversion/retroversion), but no mechanical overstrain was found (our unpublished observations). Thus, the inadequate position of the laser mark seems to be the only reason for the neck fracture in this modular system. However, prosthetic neck fractures can also occur in monobloc standard stems of contemporary design (Reigstad et al. 2008). In general, laser marks add additional risk of fracture and should thus be abandoned in connection areas of modular and fixed-neck implants. Although metal wear of about 0.6 mg per year was found in laboratory tests due to fretting in modular connections, the overall effect is negligible, as non-modular prostheses have been reported to produce about 10 mg per year alone (Viceconti et al. 1996, 1997).

The long-term results with 99% modular neck survival, 100% stem survival, and 94% cup survival after 10 and 12 years are encouraging for this modular neck dual-stem system, and similar to what has been reported in the literature (Schramm et al. 2000, Grubl et al. 2002, Aldinger et al. 2003a, b, Pieringer et al. 2006). However, the Swedish register has reported a revision rate of 21% for uncemented THA in a 12-year interval from 1992 to 2006, including all diagnoses and all revisions.

With 7% lost to follow-up, the results from our series may be interpreted as reliable (Murray et al. 1997). 17 of 173 patients underwent THA in both hips. Thus, there may be dependencies in the risk of revision between 2 primary prostheses in the same patient. However, Lie et al. (2004) found no practical difference between Kaplan-Meier curves that ignored that some patients had bilateral prostheses and curves only using the prosthesis that was inserted first or curves that were modified for the presence of bilateral prostheses.

Radiological assessment of the femoral component revealed distal cortical hypertrophy in 30% of the hips, indicating adaptive bone reactions due to mechanical changes. This may be interpreted as being a result of the proximal hydroxyapatite coating, as the incidence of cortical hypertrophy was highest in the distal region of the coating. For the Zweymueller stem, 44% of distal cortical hypertrophy was found (Grubl et al. 2002). However, this was not found in uncemented Spotorno stems (CLS) with a more proximal fixation concept (Aldinger et al. 2003a).

Femoral radiolucent lines were only found in 3% of the hips in this study, whereas CLS and Zweymueller stems had a minimum of 18% proximal radiolucent lines at the 10-year interval (Grubl et al. 2002, Aldinger et al. 2003a). However, the correlation between femoral radiolucent lines and loosening remains unclear (McLaughlin and Lee 1997).

Femoral osteolysis was rare (2%) and was only found in the proximal zones 1 and 7. This may be a sign of stress shielding, due to more distal stem fixation, and has been reported for most uncemented implants (Schramm et al. 2000, Grubl et al. 2002, Aldinger et al. 2003a, Pieringer et al. 2006). The clinical relevance is unclear, but extensive stress shielding may cause osteolysis, and thus aseptic loosening in the long term (Engh et al. 2003).

Correct femoral offset reconstruction with correct lateralization was achieved in 98% of the hips. Increased postoperative stem offset is important, since the use of press-fit cups usually causes a medialization of the center of rotation with reduced postoperative lateralization. The distance between the acetabular floor and the center line of the femur (lateralization) should remain equal pre- and postoperatively, to achieve proper joint reconstruction. Thus, the option of varus necks was helpful and was used frequently in this study.

In THA with this modular stem and 2 different pressfit cups, no cases were found with extensive polyethylene wear. 81% of the hips showed no head decentralization and 19% showed limited measurable head decentralization. Harris-Galante I cups showed more wear than EHS cups (head decentralization in 37% as opposed to 13%), which may have been a result of different polyethylene quality or liner locking mechanisms. However, a longer follow-up time for Harris-Galante I cups may also have been responsible, since EHS cups were introduced later.

The clinical results, with a median Harris hip score (HHS) of 94 points, are similar to those in other studies (Schmalzried et al. 1994, Soto et al. 2000, D'Lima et al. 2001, Kim et al. 2003). In our study, poor (7%) and moderate (14%) HHS results were mostly related to co-morbidities, associated with the high percentage of Charnley C patients (46%). Patients in Charnley class A and B had a median HHS of 94 and 95 points, respectively. The median Merle d'Aubigné score was 16 and was thus similar to the results of other authors (Tonino et al. 1995, Petsatodes et al. 2005).

No early dislocations occurred. However, 2 hips had dislocations due to cup migration with aseptic loosening, and had to be revised. One explanation for the absence of early dislocation may be reduced impingement due to better offset reconstruction with the modular stem design (Malik et al. 2007). Furthermore, reduced impingement may have been responsible for reduced polyethylene wear, less radiolucent lines, and less osteolysis in our study.

In our opinion, the use of the uncemented modular neck dual-stem system is less demanding than the use of non-modular designs. It has the potential to improve anatomical reconstruction and reduce impingement in THA. It allows adaptation of the implant to the individual patient's needs irrespective of gender, osteoporosis grade, stature, or other factors. The 10-year survival is excellent and apart from one neck fracture, no negative side effects such as fretting, wear, or extended rates of radiolucent lines, osteolysis, heterotopic ossification, and dislocation have been detected. The patients will be followed to determine the long-term outcome.
